# An Adaptive Scheme for Robot Localization and Mapping with Dynamically Configurable Inter-Beacon Range Measurements

**DOI:** 10.3390/s140507684

**Published:** 2014-04-25

**Authors:** Arturo Torres-González, Jose Ramiro Martinez-de Dios, Anibal Ollero

**Affiliations:** Robotics Vision and Control Group, University of Sevilla, Escuela Superior de Ingenieros, c/Camino de los Descubrimientos s/n, 41092 Seville, Spain; E-Mails: arturotorres@us.es (A.T.-G.); aollero@us.es (A.O.)

**Keywords:** robot-sensor network cooperation, range-only SLAM, sensor networks

## Abstract

This work is motivated by robot-sensor network cooperation techniques where sensor nodes (beacons) are used as landmarks for range-only (RO) simultaneous localization and mapping (SLAM). This paper presents a RO-SLAM scheme that actuates over the measurement gathering process using mechanisms that dynamically modify the rate and variety of measurements that are integrated in the SLAM filter. It includes a measurement gathering module that can be configured to collect direct robot-beacon and inter-beacon measurements with different inter-beacon depth levels and at different rates. It also includes a supervision module that monitors the SLAM performance and dynamically selects the measurement gathering configuration balancing SLAM accuracy and resource consumption. The proposed scheme has been applied to an extended Kalman filter SLAM with auxiliary particle filters for beacon initialization (PF-EKF SLAM) and validated with experiments performed in the CONET Integrated Testbed. It achieved lower map and robot errors (34% and 14%, respectively) than traditional methods with a lower computational burden (16%) and similar beacon energy consumption.

## 1. Introduction

This paper deals with range only SLAM, in which a robot integrates range measurements of a set of static landmarks in order to build a local map from an unknown environment and to simultaneously self-locate in that map. RO-SLAM can be very useful in a wide range of applications, such as advanced monitoring or search and rescue, in which robots cooperate with sensor nodes endowed with sensing, computing and communication capabilities. This interest has motivated the development of a growing variety of RO-SLAM methods for sensor networks [1–4], in which sensor nodes (beacons) are used as RO-SLAM landmarks. However, the great majority of these techniques consider beacons as passive landmarks, disregarding many of their capabilities.

In the last few years, several SLAM methods have been combined with active perception tools. These mechanisms evaluate different sensing actions in order to dynamically choose the action that maximizes the information acquired by the robot. Active perception tools have been used in SLAM to proactively decide on the robot motions in order to explore the environment and, hence, reduce mapping uncertainty [6–9]. The great majority of these schemes have been developed for visual SLAM and actuate only over the robot motion and/or camera orientation.

This paper presents an adaptive RO-SLAM scheme that actuates over the measurement gathering process and includes mechanisms that intentionally modify the rate and variety of range measurements that are integrated in the SLAM filter. It exploits the fact that in most cases, beacons in RO-SLAM are implemented by nodes of a sensor network, which can execute flexible *ad hoc* measurement gathering strategies. Besides the SLAM filter, which integrates all measurements gathered, the proposed scheme is based on another two main modules: the measurement gathering module and the SLAM supervisor.

The measurement gathering module enables taking and collecting direct robot-beacon, as well as measurements between beacons, called inter-beacon. It performs a controlled flooding protocol in which each beacon takes range measurements to its nearby beacons and transmits them back to the robot, naturally avoiding repeated measurements. Measurement gathering can be configured to be performed at different rates and also with different inter-beacon depth levels, so that the robot can integrate measurements of beacons that are distant from the robot's sensing zone.

Integration in SLAM of inter-beacon measurements significantly improves map estimation. Furthermore, higher gathering rates result in more accurate robot location estimation. However, both involve higher consumption of resources, such as the energy required to take and transmit measurements, the bandwidth required by the measurement collection protocol and the computational burden necessary to integrate the larger number of measurements. To cope with this, the proposed scheme includes a supervision module that monitors the SLAM performance and dynamically selects the measurement gathering settings, balancing accuracy and resource consumption.

The proposed scheme exploits the capability of the beacons of taking range measurements to other beacons and transmitting them using *ad hoc* protocols. It integrates in SLAM not only direct measurements, as traditional methods, but also inter-beacon measurements with different depth levels. Besides, it benefits from the flexibility of modifying the measurement gathering rate and inter-beacon depth level, allowing adaptation to different conditions.

The proposed scheme and its modules are general and can be applied to any SLAM filter. In this paper, it has been applied to a PF-EKF SLAM filter (described in Annex A), which is probably one of the best known and most widely researched methods. It has been validated in experiments performed in the CONET (Cooperating Objects Network of Excellence) Integrated Testbed (http://conet.us.es) [10]. In these experiments, it achieved significantly lower map and robot errors than the traditional RO-SLAM schemes with lower computational burden and similar beacon energy consumption. Besides, it showed better robustness against measurement and odometry noise levels.

The paper is organized as follows. Section 2 briefly summarizes the related work. Section 3 presents the proposed scheme and motivates it with potential applications. Section 4 describes the collection of inter-beacon measurements and their integration in the SLAM filter. Section 5 presents the SLAM supervision module. Section 6 experimentally evaluates the impact of the measurement gathering rate and inter-beacon depth level on SLAM performance and selects the parameters of the supervision module used in the experiments. Its evaluation and comparison with other schemes are in Section 7. Finally, conclusions are in Section 8.

## 2. Related Work

Range only SLAM methods rely only on range measurements. Different devices have been used to provide this kind of information, e.g., wireless sensor network (WSN) nodes [1–3] or RFID units [11,12]. Range measurements have the problem of partial observability (also present in bearing sensors): only one measurement is insufficient to constrain one location. Thus, RO-SLAM methods require the robot to move and integrate measurements from different positions in order to initialize the landmark locations. Two basic approaches have been used for landmark initialization: directly introducing the measurements using a multi-hypothesis SLAM filter (undelayed) or combining the SLAM filter with specific landmark initialization tools (delayed).

The extended Kalman filter (EKF) is probably the most commonly adopted SLAM filter. It has been combined with landmark initialization tools, such as particle filters [13], probability grids [14,15] and trilateration [2]. Trilateration methods, although simple and efficient, are very (too) sensitive to measurement noise. It was the first approach, but was soon discarded, since the SLAM performance is very dependent on the accuracy in beacon initialization. Probability grids provide better initialization, but their accuracy depends on the size and resolution of the grid. Particle filters (PFs) are maybe the most widely used landmark initialization tools in RO-SLAM. They provide better accuracy and a good number of mechanisms have been developed to reduce their computational burden.

PFs have also been combined with Rao–Blackwellised particle filters (RBPF) in works, such as [16,17]. Undelayed SLAM schemes address the multi-hypothesis problem without requiring specific initialization tools. Works in [18, 19] combined EKF with methods based on the sum of Gaussians.

The greater majority of RO-SLAM methods use only direct measurements between the robot and static beacons, disregarding the fact that most beacons can organize into sensor networks, can communicate with other beacons and can compute range measurements to other beacons. This is the case of using sensor network nodes as beacons. In recent years, the interest in schemes where robots cooperate with sensor networks has motivated the development of a number of RO-SLAM methods for sensor networks [1–3], in which sensor nodes (beacons) are used as landmarks.

Integrating in SLAM inter-beacon range measurements, *i.e.*, measurements between different beacons, involves a number of interesting advantages: they reduce map and robot estimation errors and accelerate beacon initialization. These advantages are highlighted in previous works from the authors, such as [19]. However, despite these advantages, very few methods exploiting inter-beacon measurements have been proposed. The general idea of using inter-beacon measurements was given in [14], in which different ways for incorporating inter-beacon measurements were proposed, mainly by using virtual nodes and adopting off-line map improvement using multidimensional scaling. These off-line approaches are not suitable for most applications, which require on-line map and robot locations.

In the last few years, several SLAM methods have been combined with active perception tools. These mechanisms evaluate different sensing actions that the robot can take in order to dynamically select the action that maximizes the information acquired by the robot. Active perception tools could be used in SLAM in order to accelerate the convergence of the uncertainty of an algorithm, to improve estimation accuracies or to increase the area explored by the robot. Almost all active SLAM works have been focused on visual SLAM. Davison *et al.* [6] published one of the first general active vision systems for autonomous localization, addressing issues such as uncertainty-based measurement selection, automatic map-maintenance and goal-directed steering. Vidal-Calleja *et al.* [7] actuated by moving the robot and the camera in order to optimize both localization and mapping uncertainties. Frintrop and Jensfelt [8] used an active camera to track landmarks and explore unknown environments in visual SLAM.

A very low number of active RO-SLAM methods have been reported. Merino *et al.* [20] developed a method that actuated over the steering of a robot in order to maximize uncertainty reduction in sum of Gaussians beacon initialization tools. Furthermore, almost all active SLAM schemes actuate over the robot trajectory. In fact, the term ‘active SLAM’ [21] is usually used to refer to the integration of trajectory planning in SLAM.

This paper presents a RO-SLAM scheme that actuates by configuring the measurement rate and inter-beacon depth level of the measurement gathering module. To the best of our knowledge, it is the first reported RO-SLAM method that actuates over the measurement gathering process, integrating inter-beacon measurements.

## 3. General Overview

Our work is motivated by schemes of cooperation between robots and sensor networks. Consider a GPS-denied environment, where a large number of sensor nodes have been deployed at unknown locations. For instance, they have been randomly thrown for monitoring a disaster or an accident in an industrial or urban area, where preexisting infrastructure can be damaged. The basic role of each sensor node is to periodically take measurements (e.g., toxic gas concentration), filter and compute statistics of the measurements and transmit them to a base station. For these tasks, the nodes must be endowed with sensing, communication and computational capabilities. In fact, this is the case of most commercial off-the-shelf (COTS) sensor nodes. Assume that each node is equipped with a range sensor and can measure the distance to the robot or to other nodes within its sensing zone. Again, this is not a constraint. For instance, most COTS nodes can measure the received signal strength (RSS) from incoming messages and can measure the range to the emitting node [22]. From now on, in the paper, the terms beacons and nodes will be used synonymously.

The application is to monitor the status of the event at the base station [23]. Having accurate estimations of the locations of the robot and of each node is necessary for accurate event monitoring and also enables advanced robot-sensor network cooperation strategies of interest in these problems, such as using the robot for sensor node deployment [24], replacement [25,26] or collecting data from the sensors [27], among others. GPS cannot be used. A RO-SLAM method using sensor nodes (beacons) as landmarks can be very useful for on-line estimating of node and robot locations. The robot initial pose can be used to relate this local map to global coordinates. RO-SLAM can have advantages w.r.t. visual SLAM in this problem, where suitable lighting conditions are not granted. Besides, using beacons as landmarks naturally solves data association.

**Figure 1.**
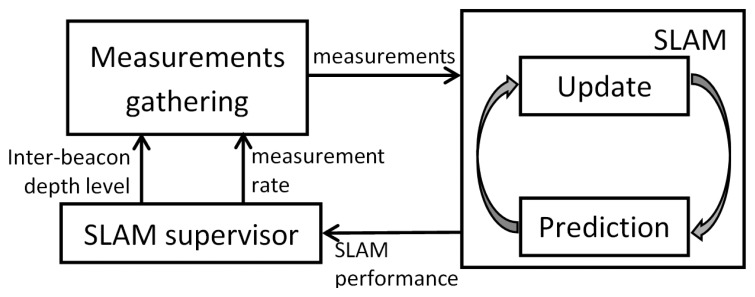
General picture of the proposed scheme.

Using sensor network nodes as landmarks can be very useful in SLAM. Nodes can measure the range to each other and collect the measurements using *ad hoc* protocols. Integrating inter-beacon measurements significantly improves the map estimation and, indirectly, the robot estimation [19]. The proposed scheme benefits from inter-beacon measurements in a dynamic and adaptive way. It employs self-adaptation mechanisms to dynamically modify the number and variety of range measurements that are integrated in the SLAM filter; see Figure 1.

The measurement gathering module is responsible for taking and collecting direct robot-beacon and also inter-beacon measurements. When a measurement gathering event is triggered, the robot starts a controlled flooding protocol in which each beacon takes range measurements to its neighbor beacons and transmits the measurements back to the robot. Measurement gathering can be configured with different inter-beacon depth levels, so that it can collect measurements of beacons that are distant from the robot's sensing zone. High inter-beacon depth levels allow for collecting more measurements and of more distant beacons than with low-depth levels. The higher the inter-beacon depth level, the higher the amount of information that can be integrated in SLAM, reducing uncertainty. In contrast, a higher inter-beacon depth level involves higher resource consumption, such as the energy used by beacons in measuring and communication, the bandwidth required for transmitting measurements and the computational burden needed to integrate measurements in the SLAM filter. Furthermore, measurement gathering can be configured to be performed at different rates involving a similar trade-off between uncertainty reduction and resource consumption.

The measurement gathering module can be configured in different modes, each of them defined by the measurement gathering rate and the inter-beacon depth level. The objective of the SLAM supervisor is to analyze the SLAM performance and select the measurement gathering mode most suitable for the current conditions.

## 4. Gathering and Integration of Inter-Beacon Measurements

This section is divided in two parts. The first one presents the gathering and collection of inter-beacon measurements. The second describes how inter-beacon measurements are integrated in the SLAM filter.

### 4.1. Measurement Gathering

Measurement gathering events are triggered by the robot and affect the static beacons in two steps: the forward stage and the backward stage. In the forward stage, static beacons perform a cascade-like measuring of range distances. The origin of the cascade is the robot, and the inter-beacon depth level (*IDL*), the number of hops of inter-beacon measurements. The backward stage orderly collects the measurements taken by all the involved beacons. All messages interchanged include a sequence number, *Seq*, that identifies the measurement gathering event. Each beacon tracks the *Seq* of the last measurement event in which it was involved. Furthermore, all messages in the forward stage include a time to live field (*ttl*), which represents the number of hops remaining until the end of the forward stage.

The forward stage starts when the robot broadcasts a forward message with *ttl* = *IDL*. Each beacon, *i*, receiving the message checks if it is a new measurement event. If it is not, the message is ignored. Otherwise, beacon *i* updates *ttl* = *ttl* − *1*, measures its distance to all the beacons in its sensing zone and buffers the measurements in *ms_i_*, the measurement set for beacon *i*. If field *ttl* > 0, the beacon broadcasts a forward message with the new *ttl*. Each beacon keeps the identifier (ID) of its parent beacon from which it received the forward message. If *ttl* = *0*, the forward message has reached its hop limit, and it is not retransmitted: the forward stage ends, and the backward stage starts.

During the backward stage, each beacon updates its own measurement set. When beacon *i* receives a backward message with suitable *Seq*, it adds the measurements contained in the message to its *ms_i_*. When the timeout of beacon *i* expires, it sends a backward message containing *ms_i_* to its parent beacon. Timeouts are set to allow orderly measurement collection.

**Figure 2.**
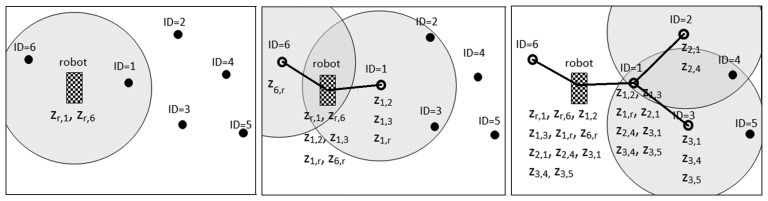
Examples of measurement gathering with: *IDL* = *0* (**left**); *IDL* = 1 (**center**); and *IDL* = 2 (**right**). Lines represent the interchange of forward (and backward) messages. Grey circles represent sensing zones of the deepest beacons for each case.

Figure 2 illustrates the operation of measurement gathering with *IDL* = 0, *IDL* = 1 and *IDL* = *2* and shows the measurements collected by each beacon in each case. With *IDL* = 0—the traditional approach where only direct robot-beacon measurements are gathered—the robot does not transmit the forward message and only collects robot-node measurements {*z_r_*_,1_,*z_r_*_,6_}, being *z_r,i_* the range between the robot and beacon *i*. With *IDL* = 1, the robot broadcasts a forward message with *ttl* = 1. Beacon *ID* = 1 receives the message, updates *ttl* = 0 and measures distances *z*_1,_*_j_*∀*j* ϵ *SZ*_1_, *SZ*_1_ being the sensing zone of beacon *ID* = 1. Since *ttl* = 0, it sends a backward message with *ms*_1_ to the robot. At the end of the backward stage, the robot has collected the following measurements: {*z_r_*_,1_,*z_r_*_,6_,*z*_1,2_,*z*_1,3_,*z*_1,_*_r_* ,*z*_6,_*_r_*}. Notice that *z_i,j_* and *z_j,i_* are not repeated measurements; they are different measurements of the same quantity. Besides, *z_i,j_* was taken by node *i* and *z_j,i_* by node *j.* The protocol obtains two measurements for each inter-beacon distance, except for the deepest beacons, of which only one is collected. Figure 2(right) shows the operation of measurement gathering with *IDL* = 2: the robot collects measurements between beacons that are far beyond its sensing zone. In this example, beacon *ID* = 1 received three forward messages. The first one was sent by the robot. It also received one from beacon *ID* = 2 and one from *ID* = 3, but they were ignored, since they had non-new *Seq* values.

The proposed protocol prevents flooding cycles, naturally avoiding repeated measurements and canceling the need for additional filtering mechanisms. Furthermore, it avoids unnecessary measurements, message transmissions and delays, improving efficiency.

### 4.2. Integration of Inter-Beacon Measurements

This section describes how inter-beacon measurements are integrated in SLAM. The adopted approach is general and can be applied to any SLAM filter in which inter-beacon measurements are integrated in a delayed way. We assume that each beacon can be in two stages: the beacon initialization stage and the core SLAM filter stage. In the first stage, the measurements are used to compute the beacon initial location using specific mechanisms, such as particle filters or occupancy grids, among others. Once the beacon has been initialized, its measurements are used to update the core SLAM filter.

Measurement gathering with *IDL* > 0 collects direct robot-beacon and also inter-beacon range measurements. The integration of measurement *z_r,__i_*, between the robot and beacon *i*, requires the observation model in Equation (13) in Annex A. The observation model for inter-beacon measurement, *r_i,j_*, between beacons *i* and *j*, is as follows:

(1) $$h_{i,j}^{IM}\left({\vec{x}}_{k}\right)=\sqrt{{\left(x_{j,k}-x_{i,k}\right)}^{2}+{\left(y_{j,k}-y_{i,k}\right)}^{2}}$$

where [*x_i_*_,_*_k_, y_i_*_,_*_k_*]*^T^* is the estimate of the location of beacon *i* at time *k.*

If beacon *i* is still in the beacon initialization stage, *z_r_*_,_*_i_* is used to initialize or update the initialization of beacon *i.* If it is in the core SLAM filter stage, *z_r_*_,_*_i_* is used in the update stage of the core SLAM filter using the following observation Jacobian:

(2) $$H_{i,k}=\left[\frac{x_{i,k}-x_{k}}{h_{r,i}},\frac{y_{i,k}-y_{k}}{h_{r,i}},0\ldots 0,\frac{x_{k}-x_{i,k}}{h_{r,i}},\frac{y_{k}-y_{i,k}}{h_{r,i}},0\ldots \right]$$

All the terms in *H_i_*_,_*_k_* are zero, except those for the entries corresponding to the robot and beacon *i.*

**Figure 3.**
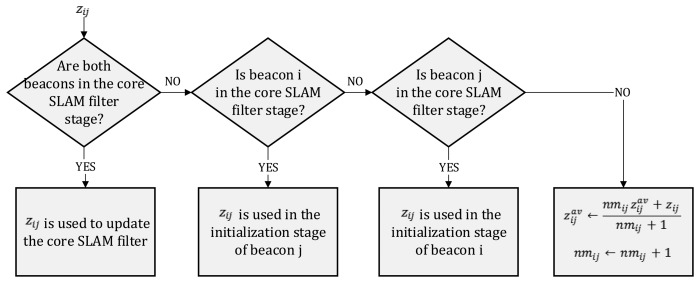
Diagram of the integration of inter-beacon measurements in the proposed scheme.

When integrating *z_i,j_*, different cases can be distinguished, depending on the stages of beacons *i* and *j;* see the summary in Figure 3. If both beacons are in the core SLAM filter stage, *z_i,j_* is used in the SLAM update stage using the following observation Jacobian:

(3) $$H_{i,j,k}^{IM}=\left[\ldots 0,\frac{x_{i,k}-x_{j,k}}{h_{i,j}},\frac{y_{i,k}-y_{j,k}}{h_{i,j}},0\ldots 0,\frac{x_{j,k}-x_{i,k}}{h_{i,j}},\frac{y_{j,k}-y_{i,k}}{h_{i,j}},0\ldots \right]$$

All the terms in $H_{i,j,k}^{IM}$ are zero, except those for the entries corresponding to beacons *i* and *j.*

If only one of the beacons, e.g., *j*, is in the core SLAM filter stage, *z_i,j_* is used in the initialization of the other beacon, e.g., *i*. If *z_i,j_* is the first measurement for beacon *i*, it is used to start the initialization of beacon *i*. For instance, in the case of using particle filters, *z_i,j_* is used to initialize *PF_i_* by deploying particles on a ring centered at [*x_j,k_, y_j,k_*]*^T^*.

If neither beacon *i* nor *j* are in the core SLAM filter stage, *z_i,j_* is kept for future use until the initialization of one of the two beacons finishes. Instead of buffering all measurements, the robot only keeps the mean value of the inter-beacon measurement— $z_{i,j}^{av}$—and the number of measurements—*nm_i,j_*. Assuming Gaussian noise of variance *R*, statistically speaking, $z_{i,j}^{av}$ is another measurement of variance *R*/*nm_i,j_*. When the initialization of beacon *i* finishes, it starts its core SLAM filter stage, and $z_{i,j}^{av}$ is used for the initialization of beacon *j*.

Therefore, when a beacon is initialized, low-variance inter-beacon measurements are integrated in the remaining beacon initialization tools, helping their convergence and originating a beacon initialization chain-reaction effect. As a result, using inter-beacon measurements drastically reduces the beacon initialization times, as will be shown in Section 6. The effect is more evident with higher values of *IDL*, since they originate a larger number of inter-beacon relations.

## 5. SLAM Supervision Module

The SLAM supervisor receives as input metrics of the SLAM performance and dynamically selects the most suitable measurement gathering mode for the current conditions. Measurement gathering with high inter-beacon depth levels allows the reducing of map initialization times and the improving of map estimation accuracy. However, once the map has been created, it is not interesting to reduce robot map error if we consider the increment in resources consumption they involve. On the other hand, changing measurement rates allows balancing between robot localization error and measurement and/or odometry noise levels. The following three measurement gathering modes can be clearly identified.

Mapping mode: The objective is to accurately initialize the map as soon as possible. When SLAM starts, the map is assumed unknown: rapidly creating an accurate estimation of the map is very important. In fact, SLAM methods for rapid map initialization have attracted significant interest in recent years, as pointed out in Section 2. Creating a map requires initializing a good percentage of the available beacons. Measurement gathering with high *IDL* drastically reduces beacon initialization times and improves beacon localization accuracy. In the *Mapping* mode, the measurement gathering is configured with high *IDL* and with the highest possible measurement rate. The *Mapping* mode should be selected while the ratio between the number of non-initialized beacons and the number of initialized beacons is above a certain threshold.

Relaxed mode: In this mode, an accurate map has already been created, and the robot can self-locate with low error. Thus, measurement gathering is configured with low rate and with *IDL* = *0* in order to save resources. The *Relaxed* mode is suitable with low odometry noise levels; the measurement rate can be reduced without impacting much on the robot location error. It is also suitable when the robot is at densely beacon-populated areas, where measurement gathering events collect a good number of measurements that allow accurate robot localization. On the other hand, the *Relaxed* mode is not suitable with high odometry noise levels, since high measurement rates should be used to compensate for the errors. The SLAM supervision module should select the *Relaxed* mode while the robot error is below a certain threshold. Of course, the ground-truth is assumed not available, and mode selection should be based on robot error estimation, as described below.

Localization mode: Its objective is to allow accurate robot self-localization in cases with higher measurement and/or odometry noise levels. The map has been already created, and measurement gathering is configured with *IDL* = *0* and a high rate, in order to improve robot localization. This mode is suitable in cases where good map and robot accuracies are desired, of course at the expense of having a higher consumption of resources than the *Relaxed* mode. The SLAM supervision module should select the *Localization* mode, while the robot error is above a certain threshold. When the robot error falls below the threshold, the mode is changed to *Relaxed* in order to save resources.

**Figure 4.**
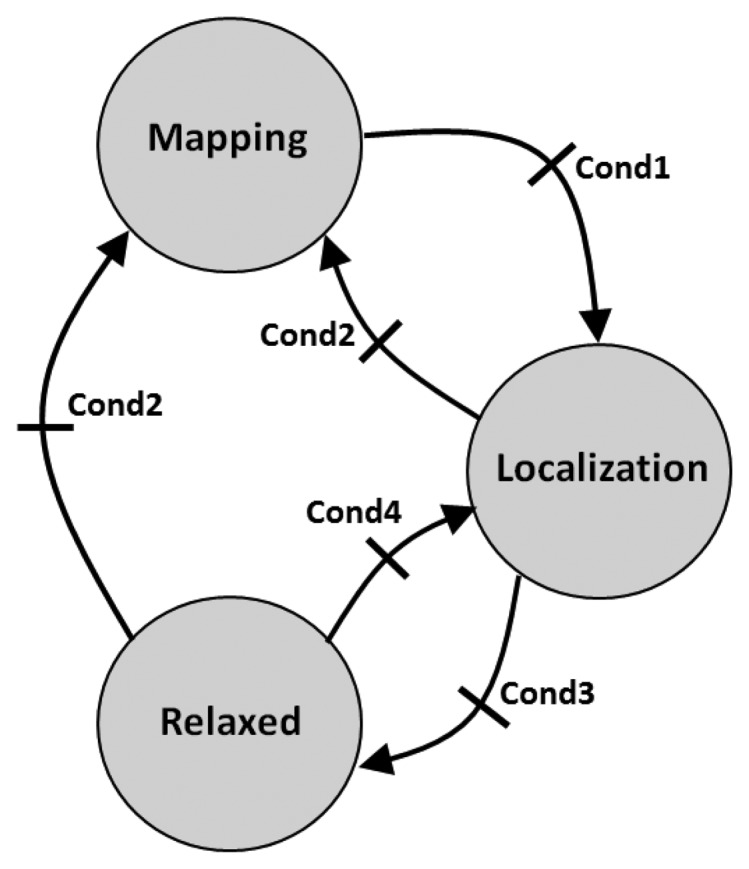
Diagram of the finite state machine implemented in the simultaneous localization and mapping (SLAM) supervisor.

In this paper, the SLAM supervision module (see Figure 4) is implemented by a simple finite state machine with three states, one per each measurement gathering mode. The robot starts at the *Mapping* state and remains at this state until *Cond1* is satisfied, *i.e.*, until the ratio between the number of non-initialized beacons and the number of initialized beacons is below threshold *T*_1_. When the map initialization finishes, the state is changed to *Localization*. Of course, at any time, the robot can move to an unexplored area where new beacons are discovered. If *Cond2* is satisfied, *i.e.*, the ratio between the number of non-initialized beacons and the number of initialized beacons is above threshold *T*_2_, the state machine changes to *Mapping*, regardless of its previous state. *Cond1* and *Cond2* are expressed as:

(4) $$\text{Cond}1:\frac{\#\text{non}‐\text{initialized beacons}}{\#\text{initialized beacons}}<T_{1}$$

(5) $$\text{Cond}2:\frac{\#\text{non}‐\text{initialized beacons}}{\#\text{initialized beacons}}>T_{2}$$

States *Localization* and *Relaxed* operate similarly. The difference is the measurement rate. The state machine stays at *Relaxed*, saving resources until *Cond4* is satisfied, *i.e.*, until the robot error is above threshold *T*_4_. In that case, the state changes to *Localization*, which involves a higher measurement rate in order to reduce the robot error. The state machine stays at *Localization* until the robot error is below *T*_3_ (*Cond3*). When *Cond3* is satisfied, it changes to *Relaxed* to save resources.

*Cond3* and *Cond4* use estimations of the robot location error. A wide variety of metrics have been used to measure the uncertainty in a statistical distribution. Some are based on the covariance matrix, while others are based on the information matrix. EKF SLAM filters use the moment representation of Gaussian distributions; hence, covariance-based metrics are more convenient. A number of metrics based on the determinant, maximum eigenvalues and trace of the covariance matrix have been developed; see, e.g., [28]. They were compared in [28] concluding that all of them perform properly.

In our scheme, the selected metric is the trace of the robot location covariance matrix. Due to the marginalization properties of the moment representation of Gaussian distributions, it is straightforward to extract the robot location covariance matrix from the EKF SLAM covariance matrix, Σ*_k_*:

(6) $$\sum_{k,\text{robot}}=\left[\begin{array}{cc} \sum_{k,11} & \sum_{k,12}\\ \sum_{k,21} & \sum_{k,22} \end{array}\right]$$

where Σ*_k,ij_* is the component in row *i* and column *j* of Σ*_k_*. Recall from Equation (10) in Annex A that the first two components of the state vector, *x⃗_k_*, are the robot location. Therefore, *Cond3* and *Cond4* are expressed as:

(7) $$\text{Cond}3:tr\left(\sum_{k,\text{robot}}\right)<T_{3}$$

(8) $$\text{Cond}4:tr\left(\sum_{k,\text{robot}}\right)>T_{4}$$

A hysteresis, *H*, is added between *T*_3_ and *T*_4_ in order to prevent ping-pong effects between states, *T*_4_ = *T*_3_ + *H*. The selection of settings and parameters of the SLAM supervisor used in the validation experiments can be found in the next section.

The proposed scheme is summarized in Algorithm 1. All the steps are detailed in Sections 4 and 5 and in Annex A.

**Algorithm 1**: The proposed scheme.
**Require**: Σ*_k_*_−1_,*μ_k_*_−1_,*u_k_*,*IDL*,*Rate*1:[
${\text{Σ}}_{k}^{-}$, 
$\mu_{k}^{-}$ ]= *EKF_Predict*(Σ*_k_*_−1_,*μ_k_*_−1_,*u_k_*), expressions (11)(12)2:{*z_k_*} = *MeasurementGathering*(*IDL*,*Rate*), in Section 43:[Σ*_k_*,*μ_k_*] = *EKF _U pdate*(
$\sum_{k}^{-}$, 
$\mu_{k}^{-}$, {*z_k_*}), expressions (14)(15)4:[*IDL*,*Rate*] = *SLAM_Supervisor*(Σ*_k_*), in Section 55:**return** Σ*_k_*,*μ_k_, IDL, Rate*


## 6. Preliminary Experiments: Evaluation of Measurement Gathering

This section presents preliminary experiments carried out to evaluate the performance of SLAM with different measurement rates and inter-beacon depth levels. The results obtained are used to select the settings and parameters of the SLAM supervisor used in the validation experiments. This evaluation was carried out using the EKF-PF SLAM described in Annex A.

**Figure 5.**
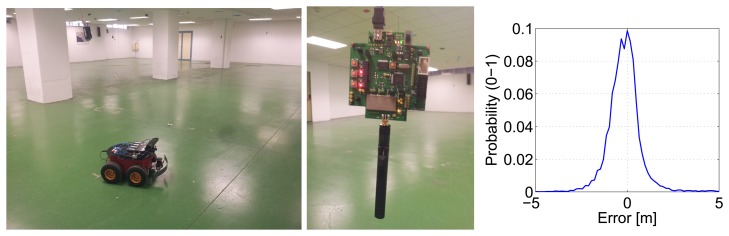
(**Left**) Picture of the CONET Integrated Testbed taken in the experiments; (**center**) Nanotron NanoPAN range sensors used in the experiments; (**right**) experimental characterization of range errors of Nanotron NanoPAN devices.

This analysis was performed using results of experiments carried out in the CONET Integrated Testbed (http://conet.us.es) [10]. That testbed is a remote tool to assess and compare methods combining robot and sensor networks; see Figure 5 (left). It is composed of Pioneer 3-AT robots, each one equipped with a Hokuyo range finder, a Microsoft Kinect, a GPS and an inertial measurement unit. The kinematic model of the robot in the experiments is the following:

(9) $$\left[\begin{array}{c} x_{k}\\ y_{k}\\ \theta_{k} \end{array}\right]=\left[\begin{array}{c} x_{k-1}+T_{k}V_{k}\sin\theta_{k-1}\\ y_{k-1}+T_{k}V_{k}\cos\theta_{k-1}\\ \theta_{k-1}+T_{k}\alpha_{k} \end{array}\right]$$

where [*x_k_, y_k_, θ_k_*]*^T^* is the robot state and *V_k_* and *α_k_* are, respectively, the odometry linear and steering velocities. *T_k_* is the differential time between *t_k_* and *t_k_*_−1_.

Among others, the CONET Integrated Testbed also includes a network of Nanotron NanoPAN range sensors; see Figure 5(center). The experimental characterization of these sensors showed that their error has zero mean and a standard deviation of σ*_m_* = 0.8 m; see Figure 5(right). Several experiments with different robot paths and beacon locations were performed and registered. Their analysis enabled extending the experiment data set with simulated experiments. A data set with a total of 120 different experiments with random paths and scenarios was generated and used in the evaluation. The ground-truth in these experiments was also logged. SLAM provides the generated map and robot locations in a local coordinate frame. For comparison with the ground-truth, an affine transform was performed on the final node locations, re-aligning the local solution into the same global coordinate frame.

This section is divided into three parts. The first two evaluate the impact of inter-beacon depth level and measurement rate on SLAM performance. The third summarizes the parameters of the SLAM supervisor used in the validation experiments.

### 6.1. Influence of Inter-Beacon Measurement Depth Level

This section analyzes the impact of inter-beacon depth level on SLAM performance and, specifically, on the beacon location errors, robot errors and beacon initialization times. The SLAM method configured with *IDL* = 0, *IDL* = 1 and *IDL* = 2 was executed with all the experiments in the data set. For brevity, the details of these preliminary experiments are obviated.

**Figure 6.**
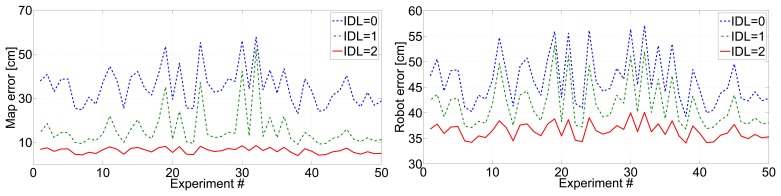
Mean beacon location errors (**left**) and mean robot location errors (**right**) in 50 experiments with *IDL* = 0, *IDL* = 1 and *IDL* = 2.

Figure 6 (left) shows the mean beacon location errors in 50 experiments with different scenarios and robot paths. Similar results were obtained in all the experiments in the data set (not shown for brevity). It can be observed that the integration of inter-beacon measurements significantly improved map accuracy: higher values of *IDL* involved lower errors. With *IDL* = 0, the mean map error had values in the range [20–60] cm. This fluctuation is originated by the sensitivity to the robot path and experiment configuration. With *IDL* = 1, map errors and their fluctuations are reduced. With *IDL* = 2, map errors were within [5–10] cm with almost no fluctuations, evidencing significant robustness to the scenario configuration. As a consequence of a more accurate map, the mean robot location errors also reduced with the value of *IDL*; see Figure 6(right). The dependence with the scenario also reduced with larger values of *IDL*, being rather uniform for *IDL* = 2. However, map error improvements with higher *IDL* were significantly larger than robot error improvements.

**Figure 7.**
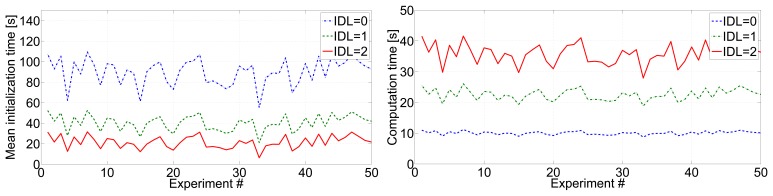
(**Left**) Mean beacon initialization times in 50 experiments with *IDL* = 0, *IDL* = 1 and *IDL* = 2; (**right**) computational times required by the robot to execute the SLAM filter in each entire experiment assuming *IDL* = 0, *IDL* = 1 and *IDL* = 2.

**Figure 8.**
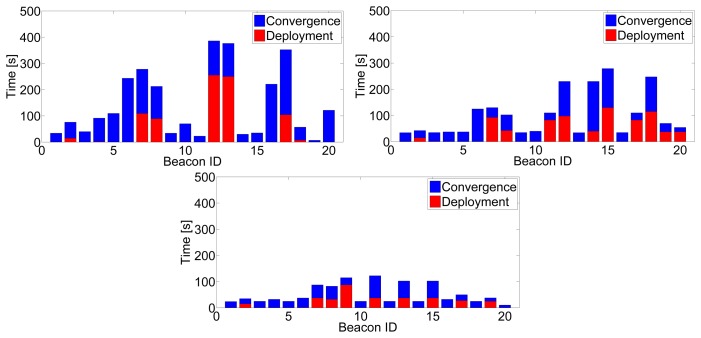
Times in which the initialization of each beacon started (red) and finished (blue) in one of the experiments with *IDL* = 0 (**top-left**), *IDL* = 1 (**top-right**) and *IDL* = 2 (**bottom**).

Integrating inter-beacon measurements also had a clear impact on beacon initialization times. Figure 7(left) shows the mean beacon initialization times for 50 experiments with different scenarios and robot paths. With larger *IDL*, a higher number of inter-beacon measurements further from the robot are integrated in the SLAM filter, resulting in lower beacon initialization times. Figure 8 shows detailed results in one of the experiments: the times in which the initialization of each beacon started (beacon discovery) are in red color, and the times in which the initialization of each beacon finished are in blue. Similar results were found in all the experiments performed. The improvement could be noticed in almost all beacons, but was particularly significant in those that were distant to the robot initial position. With *IDL* = 2 the measurement gathering protocol allows the robot to collect and integrate measurements between two beacons beyond the robot's sensing zone. Thus, the initialization of some beacons finished even before the robot took a first direct measurement for these beacons.

Of course, as a result of integrating a higher number of measurements, larger values of *IDL* involve higher resource consumption. Figure 7(right) shows the computational times required by the robot to execute the SLAM filter in each entire experiment.

Higher values of *IDL* increase the number of measurements that are integrated in the SLAM filter. Depending on the measurement noise level, increasing *IDL* could influence the SLAM estimation errors. Table 1(top) analyzes the impact on SLAM performance of changing from *IDL* = 0 to *IDL* = 1, assuming different measurement noise levels: very low (σ*_m_*_,1_ = 0.1 m), low (σ*_m_*_,2_ = 0.4 m), medium (σ*_m_*_,3_ = 0.7 m), high (σ*_m_*_,4_ = 1 m) and very high (σ*_m_*_,5_ = 1.3 m). For each case, the table summarizes the average improvements in mean map errors, mean robot errors and mean beacon initialization times in all the experiments in the data set. Values close to 0% mean no improvement. The table also shows the increment in computational burden required at the robot processor between using *IDL* = 1 and *IDL* = 0. Similarly, Table 1 (bottom) analyzes the impact of using *IDL* = 2 w.r.t. using *IDL* = 0.

As shown in Table 1, *IDL* has a deep impact on beacon initialization times. The improvements between *IDL* = 2 and *IDL* = 0 are rather steady around 72%, regardless of the measurement noise level. The improvements between *IDL* = 1 and *IDL* = 0 are around 50%. Besides, improvements between *IDL* = 2 and *IDL* = 0 are significantly more robust to measurement noise than those between *IDL* = 1 and *IDL* = 0.

*IDL* also impacts beacon location errors. The improvements in mean map error between *IDL* = 2 and *IDL* = 0 are steady around 75%, regardless of the measurement noise level. The improvements between *IDL* = 1 and *IDL* = 0 are around 63% and are more sensitive to measurement noise. Table 1 also shows that *IDL* has some influence on the robot error. The improvements between *IDL* = 2 and *IDL* = 0 are around 23%, while the improvements between *IDL* = 1 and *IDL* = 0 are around 10%. However, these improvements are low if we consider the increment in robot computational burden they involve.

As a conclusion, using *IDL* = 2 is interesting while the robot initializes the map: (1) it speeds up map initialization; (2) it reduces map error; and (3) it is rather robust to the measurement noise level and to the scenario configuration. Although *IDL* = 2 involves a large increase in the number of measurements, the increment in resources consumption can be compensated for by its shorter map initialization times. On the other hand, although using *IDL* = 1 or *IDL* = 2 can reduce robot error, they do not seem to be a convenient solution if we analyze the performance improvement versus the increment in computational costs.

### 6.2. Influence of the Measurement Gathering Rate

**Figure 9.**
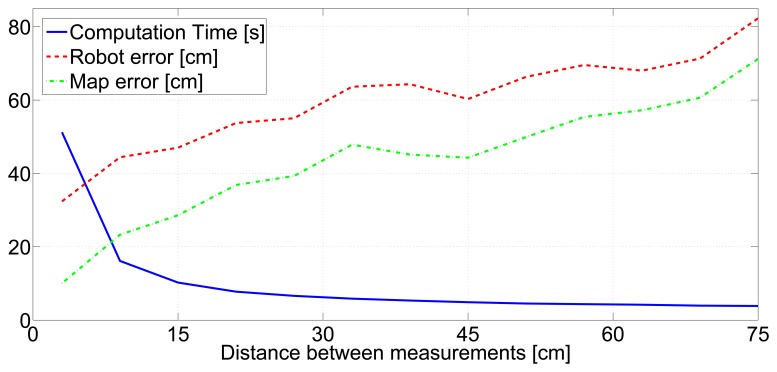
SLAM mean map error, robot error and computation times when using different measurement gathering rates.

The influence of measurement gathering rate on SLAM performance is highly dependent on the robot speed. What actually affects the SLAM performance is the spatial rate, *i.e.*, the distance between two consecutive measurement events. The SLAM method configured with *IDL* = 0 was executed in the experiments of the data set using distances between consecutive measurements in the range [5–75] cm. The tendency of the results, in Figure 9, is clear: higher rates involve lower map and robot errors, but a larger robot computational burden is required to integrate the measurements.

Figure 9 can be analyzed in terms of computational burden and SLAM accuracy. Two cases can be identified. A distance between consecutive measurements of 45 cm is a good trade-off for cases in which low resource consumption is desired and moderate map and robot errors are sufficient. Besides, the distance between consecutive measurements of 15 cm is a good trade-off for cases where higher map and robot accuracy are desired, of course, at the expense of having higher resources consumption. Both are used to define two measurement gathering rates: *Rate1*, which triggers a measurement gathering event each time the robot moves 45 cm; and *Rate2*, for which the distance between consecutive measurement events is 15 cm.

The performance of SLAM with different measurement collection rates can be impacted by the robot odometry noise level. We selected three odometry noise levels for this analysis. *O*_1_ is the odometry obtained in the real experiments performed in the CONET Integrated Testbed. *O*_2_ was obtained by adding to *O*_1_ a white noise with standard deviation σ*_o_* = 0.3. *O*_3_ was obtained by adding to *O*_1_ a white noise with σ*_o_* = 0.6. Experiments with random robot paths and the three odometry noise levels—medium (*O*_1_), medium-bad (*O*_2_) and bad (*O*_3_)—were performed. They were repeated with measurements *Rate1* and *Rate2*. Table 2 (top) shows the resulting average improvements in mean map errors, mean robot errors and mean beacon initialization times in the full experiment data set. Table 2(bottom) shows the performance evaluation between *Rate2* and *Rate1* with the different measurement noise levels analyzed in Section 6.1.

As shown in Table 2(top), the measurement rate has a significant impact on mean robot error improvements. Robot error improvements are moderate with medium odometry noise levels, but increase up to 47.89% with bad odometry *O*_3_. With good odometry, *Rate1* succeeds in obtaining good map and robot errors with low resource consumption. However, with bad odometry, the high distance between consecutive measurements in *Rate1* is insufficient to keep the robot error low. This tendency can also be noticed in the map error. However, the performance improvement between *Rate2* and *Rate1* as odometry worsens is more evident in the robot error than in the map error.

As shown in Table 2 (bottom), SLAM performance improvements are very steady, regardless of the measurement noise level. As expected, the odometry noise level and the measurement noise level have a low influence on beacon initialization times and on computational burden.

### 6.3. Configuration of the Supervisor

In SLAM, no previous knowledge is assumed: it is not possible to set in advance the measurement gathering settings. To cope with that, the SLAM supervisor analyzes the SLAM performance and dynamically modifies the measurement gathering configuration. The design of the SLAM supervisor was described in Section 5. This section selects the settings and parameters of the SLAM supervisor used in the experiments.

The objective of the *Mapping* state is to accurately estimate the map as soon as possible. Depending on the hardware used, the constraint can be the robot computational capability, the sensor nodes capability or, more rarely, the bandwidth required by the measurement collection protocol. In the experiments, *Mapping* was configured with *IDL* = 2 and *Rate2*. The state machine remains at *Mapping* until the last beacon has completed its initialization. Then, the state machine changes to *Localization*. Furthermore, if a new beacon is discovered, it changes to *Mapping*, regardless of its previous state.

In the *Localization* state, the measurement gathering module is configured with *IDL* = 0 and *Rate2*, while in the Relaxed state, it is configured with *IDL* = 0 and *Rate1*. *Cond3* and *Cond4* in Section 5 establish the transitions between *Localization* and *Relaxed*. After analyzing the preliminary experiments, the following values were selected: *T*_3_ = 0.8 and *T*_4_ = *T*_3_ + 0.3. The hysteresis between *T*_3_ and *T*_4_ was selected as *H* = 0.3. To prevent noise in robot error estimation, in the experiments, the trace of the robot location covariance matrix—Σ*_k,robot_*—was smoothed with a simple first order filter.

## 7. Experimentation and Validation

This section experimentally evaluates the proposed scheme and compares it with the following SLAM methods. **Method1** is the typical traditional SLAM scheme. During the whole experiment, it integrates only direct robot-beacon measurements (*IDL* = 0) taken at *Rate2*. **Method2** is a non-adaptable method that exploits the advantages of integrating inter-beacon measurements. It configures measurement gathering with *IDL* = 2 and *Rate2* during the whole experiment. **Method3** is an adaptable scheme that configures measurement gathering with *IDL* = 2 and *Rate2* during map initialization and, with *IDL* = 0 and *Rate1*, once the map has been initialized. Table 3 summarizes their configuration.

It should be noticed that all these methods could be very easily implemented by the proposed scheme simply by changing the state transitions parameters in the SLAM supervisor. For instance, *Method1* could be implemented by enforcing that the finite machine is always at state *Localization*. Furthermore, *Method3* is a particular case of the proposed scheme with only states *Mapping* and *Relaxed*. From this point of view, our method generalizes existing techniques, adding flexibility and reactivity.

The validation experiments were performed in the CONET Integrated Testbed. A validation data set similar to that in Section 6 was created with 80 different robot paths and scenario configurations. The Nanotron NanoPAN range sensors were configured with a maximum sensing range of 8 m. All the methods were executed in exactly the same conditions: all are based on the PF-EKF scheme described in Annex A and use the same EKF and PF parameters.

### 7.1. Evaluation and Comparison

**Figure 10.**
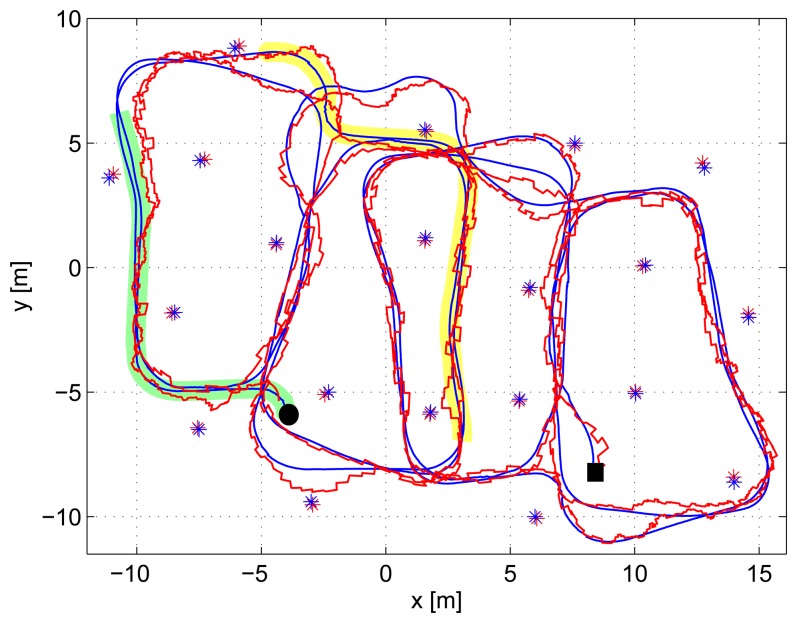
Results of the proposed SLAM scheme in one experiment. Estimations are in red and the ground-truth in blue.

**Figure 11.**
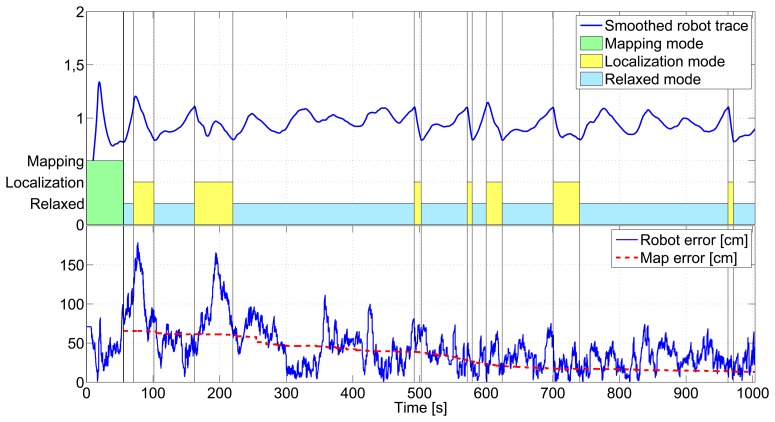
Details of the operation of the proposed scheme along the experiment: (**top**) values of the smoothed robot trace; (**center**) the measurement gathering state; (**bottom**) robot and map location errors.

Figure 10 shows the result of applying the proposed SLAM scheme in one experiment. Ground-truth robot and beacon locations are in blue, while the resulting estimations are in red. The robot initial and final locations are marked in the figure with a black circle and a black rectangle, respectively. Figure 11 details its operation during the execution of the experiment. It shows: (top) the values of the smoothed robot trace used as robot error estimation; (center) the measurement gathering state provided by the SLAM supervisor; and (bottom) the robot and map location errors at each time, represented in blue and red, respectively.

It starts at *t* = 0 s at state *Mapping* and remains at that state; see Figure 11, until the particle filters of all the discovered beacons converge, which occurred at *t* = 55.6 s. Figure 10 shows in green the path followed by the robot while the scheme is at the *Mapping* state. Due to the sensors' maximum sensing range, the robot was capable of integrating direct robot-beacon measurements only from 55% of the 20 beacons deployed in the scenario. The particles filter of the beacons distant from the robot's sensing range (45% of the beacons) converged using only inter-beacon measurements. As expected, the *Mapping* state, with *IDL* = 2 and *Rate2*, rapidly created an accurate map. At *t* = 55.6 s, the last PF converged; the map was initialized with a map error of 65.5 cm, and the state machine changed to *Localization*. From that time on, the map error is represented in Figure 11.

After the *Mapping* state, the smoothed robot trace was very low (meaning accurate estimation), fulfilling *Cond3*, and thus, when the last PF converged, the state machine changed to *Localization* and immediately changed again to *Relaxed*.

The state machine remained at *Relaxed* until t = 70.4 s, in which the smoothed robot trace grew above *T*_4_, satisfying *Cond4*: the state machine changed to *Localization*. The robot trace grew, because the beacons that surrounded the robot at those times had significantly high errors, higher than the mean map error. Integrating their measurements increased the robot error. After changing to the *Localization* state, the increase in the number of measurements integrated in SLAM (originated by changing from measurement rate from *Rate1* (*Relaxed*) to *Rate2* (*Localization*)) rapidly decreased the smoothed robot trace. At *t* = 101 s, it fell below *T*_3_, fulfilling *Cond3*, and the state changed again to *Relaxed*.

The *Relaxed* state remained until *t* = 162 s. The robot motion had two consecutive curves, which suddenly degraded the robot odometry. This degradation in odometry originated a sudden increase in the robot trace. At *t* = 162 s, the smoothed robot trace became above *T*_4_, fulfilling *Cond4*, and the state machine changed to *Localization*. With a higher measurement gathering rate, the robot location uncertainty improved; and at *t* = 219.8 s, the smoothed robot trace fell below *T*_3_, and the state changed again to *Relaxed*.

In the rest of the experiment, the smoothed robot trace increased and reduced as the robot followed its path. The state was changed when necessary reacting and adapting to the changing conditions. At the end of the experiment, the robot and map errors w.r.t. the ground-truth were, respectively, 32.4 cm and 14.3 cm, enough for a wide variety of applications. In the full experiment, the state machine was at the *Mapping* state during 7.60% of the total time, at *Localization* during a total of 17.24% and at *Relaxed* during a total of 75.16%. It was at the *Relaxed* state during the greater majority of the time involving the low consumption of resources. The same experiment was repeated, obtaining similar results. A video illustrating another experiment can be seen at [30].

**Figure 12.**
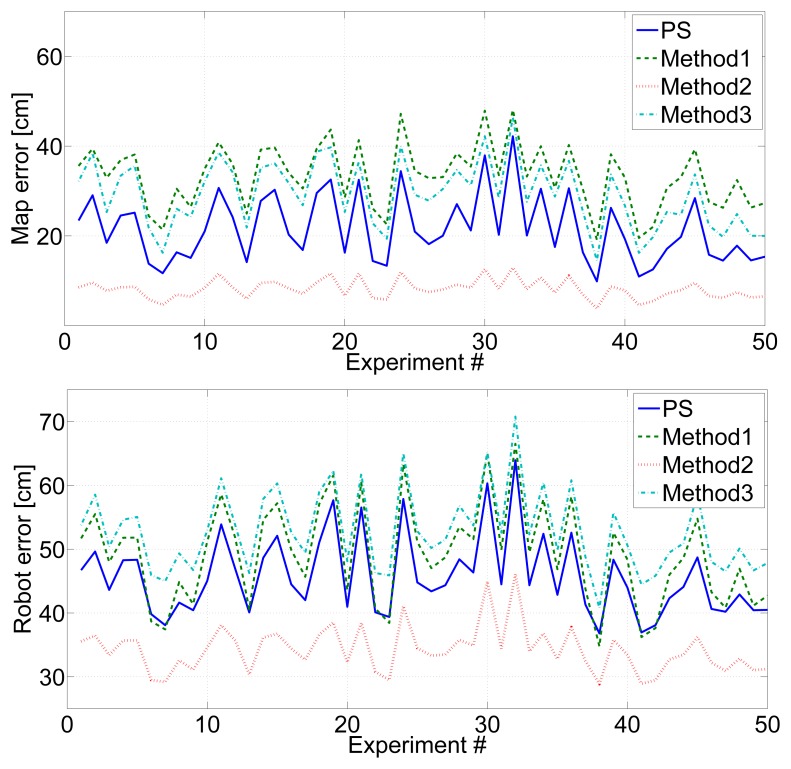
Mean beacon location errors (**top**) and mean robot location errors (**bottom**) in 50 experiments performed with *Method1, Method2, Method3* and the proposed scheme (PS).

**Figure 13.**
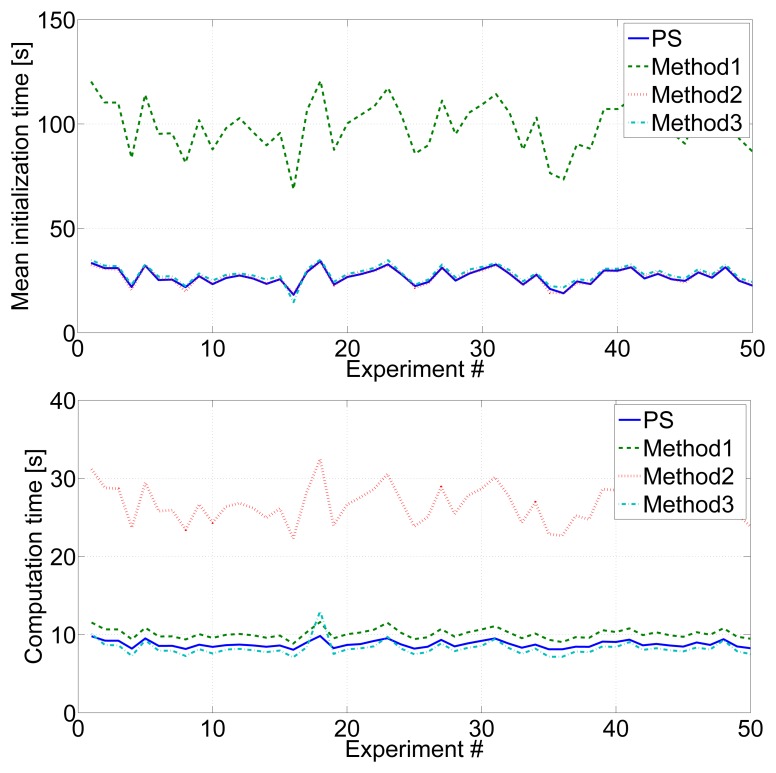
Mean beacon initialization times (**top**) and computational times (**bottom**) required in each of the 50 experiments performed with *Method1, Method2, Method3* and the proposed scheme (PS).

In the following, the proposed scheme (PS in the figure) is compared with *Method1, Method2* and *Method3*. All the methods were executed with each experiment in the validation data set with exactly the same parameters. Figure 12 shows the mean beacon location errors (top) and mean robot location errors (bottom) in 50 experiments. Figure 13 shows the mean beacon initialization times (top) and the computation times required (bottom) in each of the 50 experiments.

*Method2*, which uses *IDL* = 2 and *Rate2* during the whole experiment, obtained the lowest map and robot mean errors. *Method1*, which uses *IDL* = 0 and *Rate2* during the whole experiment, obtained the worst map and robot errors. *Method3* and the proposed method obtained intermediate map and robot errors. The errors in the proposed method were lower than in *Method3*. Besides the *Mapping* state, which is the same for both, the proposed method has two states, *Localization* and *Relaxed*, that allow flexibility to balance between accuracy and burden, while *Method3* only has one state. The *Localization* state enables the proposed method to reduce errors when necessary, involving very low extra costs.

*Method2, Method3* and the proposed method had similar mean beacon initialization times, since they all operate with *IDL* = 2 and *Rate2* during map initialization. In contrast, *Method1* uses *IDL* = 0 obtained beacon initialization three times higher. The robot computational burden of *Method2* was three times higher than that of *Method1, Method3* or the proposed method. It should be noticed that both, *Method3* and the proposed scheme, require lower robot burden than *Method1*, the traditional method. Although both consume more resources during map initialization, they rapidly obtain an accurate map estimation that allows them to be at the *Relaxed* state, saving resources, during large periods of the experiment. As a result, in total, *Method3* and the proposed scheme obtain lower robot and map errors with lower burden than *Method1*. Furthermore, the computational burden of the proposed method is a bit higher (6.5%) than that of *Method3*, since the proposed scheme goes in the *Localization* state from time to time in order to the reduce robot error, while *Method3* keeps at the *Relaxed* state during all the time after map initialization.

*Method2* obtained the lowest map and robot mean errors, but required too high of a robot computational burden. The best tradeoffs between performance and burden were *Method3* and the proposed method (PS). Both overtook Method1. In particular, the proposed method had lower map (34%) and robot error (14%) than *Method1*, requiring also lower computational burden (16%). When comparing with *Method3*, the proposed method had lower map and robot error than *Method3* (19% and 31%, respectively) requiring only a bit higher computational burden (6.5%).

The proposed scheme is also efficient in terms of the energy consumed by the beacons. We obtained a consumption model using the information provided by the manufacturer. The radio module is responsible for the greater part of the consumption. Each measurement takes 12 ms, during which, the emitter and the receiver interchange the measurement request and response packets. Both beacons transmit during 6 ms (consuming 210 mA) and receive during 6 ms (consuming 51 mA). The consumption of both beacons is the same and depends on the number of measurements in which they are involved. Table 4 shows the mean number of measurements in which beacons are involved in the 50 experiments analyzed. It also shows the mean beacon energy consumptions in these experiments. As expected, *Method2* consumed 360% more than the rest. The consumption of the proposed scheme was a bit higher than *Method1* (3.9%) and than *Method3* (1.7%). However, these small differences are compensated by the significant improvements in accuracy.

### 7.2. Robustness

This section experimentally evaluates the robustness of the proposed scheme against measurement and odometry noise levels using the experiments in the validation data set. The measurements and odometry with different noise levels were obtained by adding noise to the real measurements and odometry as in Section 6.

**Figure 14.**
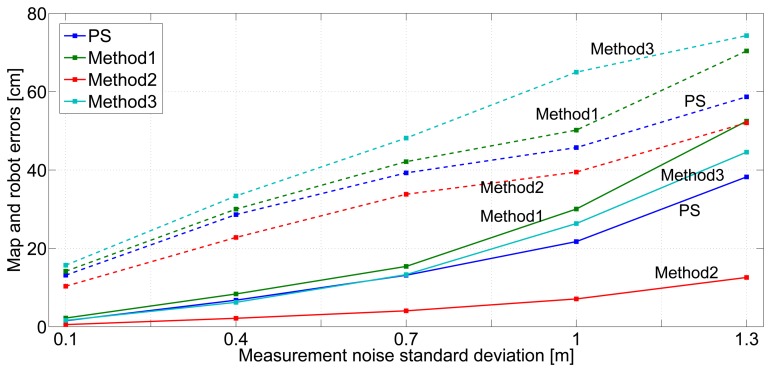
Robustness of *Method1, Method2* and *Method3* and the proposed scheme (PS) against measurement noise level: the mean robot errors are in full lines, while the mean map errors are in dashed lines.

Figure 14 shows the robustness in the mean map error (full lines) and robot error (dashed lines) against measurement noise with standard deviation in the range [0.1–1.3] m. *Method2* exhibited the best robustness both in map and robot errors. It uses *IDL* = 2 and *Rate2* and integrates a very high number of measurements, achieving good robustness to measurement errors. *Method1* and *Method3* obtained the worst robustness. *Method1* integrates only direct measurements, lacking the relations established by inter-beacon measurements and being more sensitive to measurement errors. In *Method3*, once the map has been initialized, measurement gathering is configured with *Rate1*, which is rather sensitive to measurement errors.

The proposed scheme has moderate robustness against measurement noise. It is more robust than *Method1* and than *Method3*. It includes the *Localization* state, designed to obtain good accuracy in the presence of high measurement and/or odometry noise levels. This is why it is more robust than *Method3* when estimating robot location. Besides, its advantages over *Method3* in map error are more evident with higher measurement noise levels (standard deviations higher than 0.7 m).

**Figure 15.**
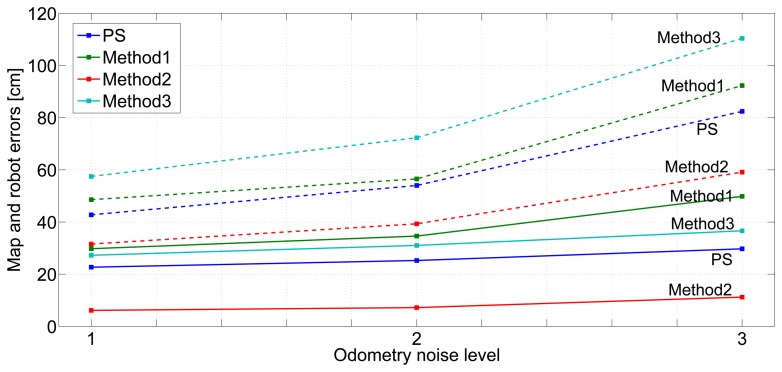
Robustness of *Method1, Method2* and *Method3* and the proposed scheme (PS) against odometry noise level: the mean robot errors are in full lines, while the mean map errors are in dashed lines.

Figure 15 shows the robustness of the mean map error (full lines) and mean robot error (dashed lines) when using the odometry, *O*_1_, *O*_2_ and *O*_3_, defined in Section 6. Odometry *O*_1_ corresponds to “1” in the abscissa axis. *O*_2_ and *O*_3_ correspond to “2” and “3”. *Method3* had the worst robustness against odometry noise. After map initialization, *Method3* always uses *Rate1*, which is very sensitive to odometry noise. The best robustness was obtained with *Method2*. On the other hand, although the odometry noise level has a moderate influence on map error, *Method1* is sensibly less robust than the rest: the rest of the methods integrate inter-beacon measurements during map initialization, making map estimation more robust to odometry noise.

*Method2* has the best robustness, but, as analyzed in Section 7.1, it involves a very high computational burden. *Method1* and *Method3* have poor robustness. The proposed scheme has better robustness than *Method1* and than *Method3*.

## 8. Conclusions

This paper presents a RO-SLAM scheme that actuates over the measurement gathering process. It includes a measurement gathering module that implements a protocol by means of which it can take and collect at configurable rates direct robot-beacon and inter-beacon range measurements of configurable depth levels. It also includes a supervision module that monitors the SLAM performance and dynamically selects the configuration of the measurement gathering module. The proposed scheme exploits the capability of beacons of taking range measurements and transmitting them using *ad hoc* protocols. Besides, it exploits the flexibility of dynamically modifying the measurement gathering rate and inter-beacon depth level, allowing its adaptation to changing conditions. To the best of our knowledge, it is the first active RO-SLAM method that actuates over the measurement gathering process integrating inter-beacon measurements.

The SLAM supervision module implements a simple finite state machine with three states, *Mapping, Localization* and *Relaxed*, each of them with different inter-beacon depth levels and measurement rates. The SLAM performance metrics selected are the percentage of discovered, but not initialized, beacons and the estimation of the robot error using the trace of the robot location covariance matrix.

The proposed scheme and its modules are general and can be applied to any SLAM filter. In this paper, it has been applied to a PF-EKF SLAM filter, which is maybe one of the most common RO-SLAM methods. It has been validated in simulations and experiments performed in the CONET Integrated Testbed, and its performance has been compared to the traditional methods based on integrating only direct robot-beacon measurements. The experiments show how the SLAM supervision module dynamically changes the state, changing the configuration of measurement gathering, impacting on which measurements are integrated in the SLAM filter. Its evaluation evidences the advantages of its adaptation and configuration capabilities. In these experiments, it obtained map and robot location errors significantly lower (e.g., 34% and 16%, respectively) than traditional RO-SLAM techniques, requiring 16% lower computational burden and similar beacon energy consumption. Besides, it exhibited better robustness against measurement and odometry noise levels.

The proposed scheme allows high flexibility, being able to implement a high number of methods by simply modifying the parameters of the SLAM supervisor. Thus, it can be easily adapted to particular problems or applications. This work opens wide fields for research. The SLAM supervisor used in this paper is implemented by a simple finite state machine with only three states. More complex SLAM supervisors could be devised in order to enable more refined adaptation to concrete conditions.

## Figures and Tables

**Table 1.** SLAM performance evaluation between *IDL* = 1 and *IDL* = 0 (**top**) and between *IDL* = 2 and *IDL* = 0 (**bottom**).

***IDL*** = **1** *vs.* ***IDL*** = **0**	σ*_m_*_,1_	σ*_m_*_,2_	σ*_m_*_,3_	σ*_m_*_,4_	σ*_m_*_,5_
Improvement in mean map error	69.60%	65.53%	61.16%	63.21%	64.81%
Improvement in mean robot error	9.1%	9.99%	8.19%	10.21%	11.56%
Improvement in mean beacon initialization time	61.41%	57.25%	59.1%	48.94%	40.24%
Increase in mean computational burden	46.43%	55.51%	64.74%	71.38%	104.15%

***IDL*** = 2 *vs.* ***IDL*** = **0**	σ*_m_*_,1_	σ*_m_*_,2_	σ*_m_*_,3_	σ*_m_*_,4_	σ*_m_*_,5_
Improvement in mean map error	76.22%	74.37%	73.70%	75.39%	75.04%
Improvement in mean robot error	27.06%	24.07%	19.76%	21.35%	26.08%
Improvement in mean beacon initialization time	75.60%	74.51%	71.3%	71.7%	69.54%
Increase in mean computational burden	108.63%	119.51%	140.11%	151.27%	183.12%

**Table 2.** SLAM performance evaluation between *Rate2* and *Rate1* with different odometry levels (**top**) and with different measurement noise levels (**bottom**).

***Rate2*** *vs.* ***Rate1***	*O*_1_	*O*_2_	*O*_3_
Improvement in mean map error	31.58%	38.61%	48.64%
Improvement in mean robot error	24.80%	35.43%	47.89%
Improvement in mean beacon initialization time	6.99%	12.53%	8.01%
Increase in mean computational burden	113.66%	121.96%	120%

***Rate2*** *vs.* ***Rate****1*	σ*_m_*_,1_	σ*_m_*_,2_	σ*_m_*_,3_	σ*_m_*_,4_	σ*_m_*_,5_
Improvement in mean map error	30.16%	30.50%	32.97%	31.40%	33.82%
Improvement in mean robot error	24.45%	21.96%	25.56%	24.58%	28.65%
Improvement in mean beacon initialization time	3.07%	6.89%	6.49%	11.72%	21.97%
Increase in mean computational burden	135.98%	141.5%	134.35%	135.12%	139.20%

**Table 3.** Description of the methods' configuration.

	**During Mapping**	**After Mapping**
**Method1**	*IDL* = 0, *Rate2*	*IDL* = 0, *Rate2*
**Method2**	*IDL* = 2, *Rate2*	*IDL* = 2, *Rate2*
**Method3**	*IDL* = 2, *Rate2*	*IDL* = 0, *Rate1*
**Proposed scheme (PS)**	*IDL* = 2, *Rate2*	*IDL* = 0, *Rate1/Rate2*

**Table 4.** Comparison of beacon energy consumption of *Method1, Method2, Method3* and the proposed scheme during the experiments.

	***Proposed Scheme***	***Method1***	***Method2***	***Method3***
# of measurements involving a beacon	1,830	1,760	8,300	1,800
Mean beacon energy consumption (J)	11.53	11.09	52.29	11.34

## A. Appendix A

### PF-EKF SLAM in a Nutshell

This annex briefly summarizes the PF-EKF SLAM filter used in the experiments. The proposed scheme can be applied to any SLAM filter. This is why it has been preferred to present the well-known PF-EKF SLAM filter in a condensed way. For a more detailed description, refer to [29].

EKF-SLAM is a well-known technique that recursively solves the online SLAM problem where the map is feature-based. It estimates, at the same time, the robot pose and the position of the beacons. Thus, the state vector adopted is:

(10) $${\vec{x}}_{k}={\left[rp_{k},x_{1,k},y_{1,k},\ldots ,x_{n,k},y_{n,k}\right]}^{T}$$

where *rp_k_* is the robot pose and [*x_i,k_*,*y_i,k_*]*^T^* is the 2D location of static beacon *i* at time *k*. The robot pose is usually taken as *rp_k_* = [*x_k_, y_k_, θ_k_*]*^T^*, the robot 2D position and heading at time *k*.

During the EKF-SLAM prediction phase, the mean *μ_k_* and covariance Σ*_k_* of the state vector *x⃗_k_* are updated as follows:

(11) $$\mu_{k}^{-}=f\left(\mu_{k-1},u_{k}\right)$$

(12) $$\sum_{k}^{-}=A_{k}\sum_{k-1}A_{k}^{T}+Q_{k}$$

where *Q_k_* is the covariance matrix that models the uncertainty in *f*(*x⃗_k_*_−1_,*u_k_*), the robot state transition model. *f* is often non-linear, and its Jacobian $A_{k}=\frac{\partial f}{\partial {\vec{x}}_{k-1}}$ is used.

When the robot takes at time *k* a new robot-beacon range measurement, *z_r,i_*, from beacon *i*, the update phase of the EKF can be performed using the observation model, *h_i_*, which expresses the distance between the robot and beacon *i:*

(13) $$z_{r,i}=h_{i}\left({\vec{x}}_{k}\right)=\sqrt{{\left(x_{k}-x_{i,k}\right)}^{2}+{\left(y_{k}-y_{i,k}\right)}^{2}}$$

*R_i_*_,_*_k_* is the variance of the uncertainty in *h_i_*(*x⃗_k_*). Hence, the mean and covariance of the state can be updated as:

(14) $$\mu_{k}=\mu_{k}^{-}+K_{k}(z_{r,i}-h_{i}(\mu_{k}^{-}))$$

(15) $$\sum_{k}=\left(I-K_{k}H_{i,k}\right)\sum_{k}^{-}$$

where *H_i_*_,_*_k_* is the Jacobian of *h_i_* at time *k* and *K_k_* is the Kalman gain.

In PF-EKF SLAM, the partial observation problem is solved using particle filters (PFs) for beacon initialization. As analyzed in Section 2, PFs are one of the most widely used beacon initialization tools, due to their flexibility and robustness. PFs can represent any probability distribution and can naturally solve the partial observation problem, due to their multi-hypothesis capability.

The integration of PFs and the EKF is as follows. When the robot receives the first range measurement, *z_r,__i_*, from beacon *i*, it initializes an auxiliary PF for beacon *i, PF_i_*, in which the particles, which are hypotheses of the beacon location, are spread around the robot at distances drawn from an annular distribution centered at the robot current location with mean *z_r,__i_* and a width that depends on the measurement variance. When the robot receives a new measurement from beacon *i*, the particles of *PF_i_* are updated, making them condensate towards the beacon position. PFs implement the so-called importance resampling, which draws *M* particles with replacement. The probability of drawing each particle is given by its importance weight. Importance resampling helps to have a faster convergence of the PFs. We considered that a PF has converged in a Gaussian distribution when the covariance of its particles is lower than a certain value. In the experiments shown in this paper, PFs were assumed with 300 particles, and importance resampling replaced *M*=10% of the particles.

When *PF_i_* converges, the beacon location, [*x_i_*,*y_i_*]*^T^*, is computed as the weighted mean of all particles, and it is added to the EKF state vector, *x⃗_k_*. Thus, along the PF-EKF SLAM iterations, each beacon can be in two stages: the PF stage and the EKF stage. When the robot receives a measurement from a beacon, it will be used to update its PF or the EKF, depending on the beacon stage. The flexibility of PFs is at the expense of computational burden. PF iterations require significantly more burden than EKF iterations. That is why PF convergence is often taken as a metric for computational cost in PF-based SLAM methods.
